# What's new in emergencies, trauma, and shock? Role of simulation and ultrasound in acute care

**DOI:** 10.4103/0974-2700.41779

**Published:** 2008

**Authors:** Fatimah Lateef

**Affiliations:** Department of Emergency Medicine, Singapore General Hospital and Yong Loo Lin School of Medicine, National University of Singapore, Singapore. E-mail: fatimah.abd.lateef@sgh.com.sg

## SIMULATION AND ULTRASOUND EDUCATION IN ACUTE CARE

### Simulation

The field of medical simulation is expanding and evolving rapidly. Early simulators focused on patients, and physicians had to practice on these volunteer patients. Other types of simulators have focused on diseases and the response to clinical intervention. Active models which attempt to reproduce living anatomy and physiology were more recent developments. For example, the famous “Harvey” manikin is able to recreate many of the physical findings of the cardiology examination, using palpation, auscultation, and electrocardiography. More recently, interactive models which respond to action taken by the physician or student have been developed.[1]

Computer simulations have the advantage of allowing the trainee to make judgment and also correct errors. This process of iterative learning through assessment, evaluation, decision making, and error correction creates a much stronger learning environment than passive instruction. Current technology enable simulators to present complex, interactive, and lifelike experiences that assist in medical education. These lifelike simulations are expensive, but the ‘classroom of the future’ would probably contain several types of simulators, in addition to textual and visual-learning tools. This educational environment will allow students to enter the clinical years better prepared and with a higher skill level. For the advanced or postgraduate student, they will have a more concise and comprehensive method of training and retraining, incorporating new clinical procedures into their skill set. This can assist the process of credentialing and competency evaluation which is a major task for regulatory bodies and medical institutions. This classroom of the future too can form the basis of a clinical skills unit for continuing education of medical personnel. Similar to the use of periodic flight training for airline pilots, this unit can assist practitioners throughout their career.

### Ultrasound education and training

Ultrasound education has evolved over the last 30 years. Early teaching programs in ultrasound followed a traditional apprenticeship model of “see one, do one, teach one.” These programs were usually hospital-based. As the discipline progressed, many programs developed an academic base with early exposure to clinical patients. Now, using the ultrasound educational simulator, clinical experience can begin even earlier. Simulator-based education is also ideally suited for incorporation in distance learning and continuing medical education. The modern ultrasound simulator uses real scans to overcome the limitations posed with schematic or cartoon-like images. This makes it more realistic with freedom of movement, random acimages, and lifelike scanning techniques. Machine controls and operator settings function in real time, thus allowing trainees to make mistakes and correct them. Trainees can learn ‘knobology,’ sonographic anatomy and eye-hand coordination. Once a basic level of achievement is attained, they can proceed to a more advanced level. The knowledge of basic structure, appearance and pattern recognition, and the range of ‘normal variation’ can be repeated until it is mastered.[2]

## ASSESSMENT OF CLINICAL COMPETENCE

**Figure 1:**
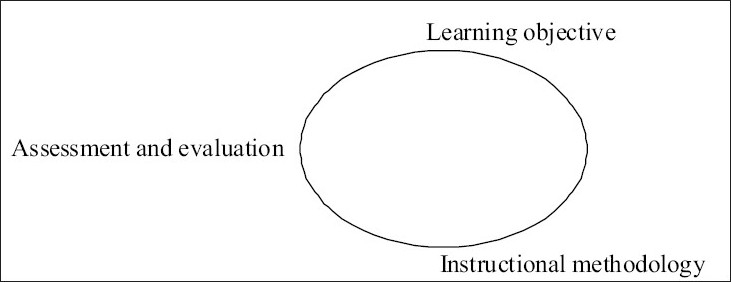
The learning cycle

This is best done with a multifaceted approach, focusing on components of knowledge, practical skills, and attitudes. Competence refers to the ability to carry out a set of tasks or roles adequately and effectively. Competency-based education must place greater emphasis on the attainment of required competency and practical skills in the “real” practice scenario or environment. Facts studied must be integrated and made relevant to clinical practice, and often it is useful to follow the learning cycle below[3–5] [Figure 1].

## ULTRASOUND IN EMERGENCY AND ACUTE CARE

Ultrasound has now become an integral part of emergency medicine and acute care. It is routinely used in Level 1 Trauma Centers and many emergency departments (EDs). It also represents part of the Advanced Trauma Life Support (ATLS) guidelines for diagnostic tool in trauma patients.[6]

Emergency ultrasound specifically refers to an examination performed by an emergency physician at the bedside, with limited goals that will allow for rapid decision making, identification of life-threatening diagnoses, and the ability to expedite emergency management. This ultrasound is different from the one done by the radiology technician, radiologist, or cardiologist.[7–9]

Some examples of the range of cases where emergency ultrasound is utilized include:

- Detection of intraperitoneal bleed in trauma,
- Diagnosis of pericardial effusion or tamponade,
- Detection of abdominal aortic aneurysm,
- Cholecystitis/cholelithiasis,
- Hydronephrosis, and
- In the assessment of abdominal pain.

The term Focused Assessment with Sonography for Trauma (FAST) was first coined in 1996 and has persisted as the accepted acronym for trauma ultrasound examination. It comprises of the four-view examination: perihepatic, perisplenic, pelvic, and pericardial view. This rapid, noninvasive and practical nature of ultrasound for bedside evaluation of critically injured patients has indeed changed the evaluation of blunt abdominal trauma.[10]

Today, more and more procedures are being performed under ultrasound guidance as there is increasing evidence they are more efficient and safer than conventional techniques.[11] Some examples of this include:

- Use in incision and drainage of superficial abscess. Ultrasound of suspected areas can be used to localize a fluid collection accurately and guide aspiration or incision and drainage.
- Management of peritonsillar abscesses, where intraoral abscesses have been found to be rather well tolerated. As the anatomy of the area is complex, a high frequency curvilinear intracavity probe covered with a sterile sheath is used for the examination.
- Ultrasound has been used to guide various joint aspirations/arthrocentesis. It is particularly helpful when initial blind aspiration has failed or when patient characteristics such as obesity makes the procedure technically difficult.
- Suprapubic bladder aspiration.
- *Thoracocentesis*. Ultrasound allows for more rapid detection and safer percutaneous drainage of pleural effusions than conventional methods and
- *Pericardiocentesis*. Echocardiography is sensitive for the diagnosis of pericardial effusion and tamponade. On ultrasound, pericardial effusion is seen as a dark or anechoic space between the heart and pericardium.

Many invasive procedures are now safer and more efficient with the use of ultrasound guidance.[12–16]

Newer areas of use include detection of testicular masses and torsion, ocular ultrasound, and musculoskeletal applications. Perhaps in future the technique can also be used more frequently in prehospital care.

## CONCLUSION

Future research in trauma ultrasound will likely focus on integration with current diagnostic and imaging modalities. Highly portable ultrasound units will also become more popular, including the evaluation of transducer frequency for parenchymal injuries. More large multicenter trials will be required to evaluate previous hypotheses and clarify issues such as scoring systems and clinical pathways. Emergency physicians and trauma specialists will continue to be on the forefront of this work. The role of ultrasound in pediatric trauma too needs to be better defined and clarified.

Thus, ultrasound is certainly here to stay and the availability of simulation ultrasound training is a necessary step toward the future.
